# Tentative exploration of pharmacodynamic substances: Pharmacological effects, chemical compositions, and multi-components pharmacokinetic characteristics of ESZWD in CHF-HKYd rats

**DOI:** 10.3389/fcvm.2022.913661

**Published:** 2022-09-14

**Authors:** Li-li Hong, Yan Zhao, Wei-dong Chen, Chen-yu Yang, Guo-zhuan Li, Hong-song Wang, Xiao-yu Cheng

**Affiliations:** ^1^School of Pharmacy, Anhui University of Chinese Medicine, Hefei, China; ^2^Anhui Province Key Laboratory of Chinese Medicinal Formula, School of Pharmacy, Anhui University of Chinese Medicine, Hefei, China; ^3^Synergetic Innovation Center of Anhui Authentic Chinese Medicine Quality Improvement, School of Pharmacy, Anhui University of Chinese Medicine, Hefei, China; ^4^MOE-Anhui Joint Collaborative Innovation Center for Quality Improvement of Anhui Genuine Chinese Medicinal Materials, School of Pharmacy, Anhui University of Chinese Medicine, Hefei, China; ^5^Department of Geriatric Cardiology, First Affiliated Hospital of Anhui University of Chinese Medicine, Anhui University of Traditional Chinese Medicine, Hefei, China

**Keywords:** Ershen Zhenwu Decoction, chronic heart failure with heart-kidney Yang deficiency, UPLC/Q-TOF-MS, pharmacokinetic, pharmacodynamic substances

## Abstract

The chemical components of Xin'an famous prescription Ershen Zhenwu Decoction (ESZWD) are still unclear. The results showed that ESZWD could significantly reduce left ventricular end diastolic diameter, decrease N-terminal pro-brain natriuretic peptide (NT-proBNP), angiotensinII, aldosterone, reactive oxygen species, and malondialdehyde, increase serum superoxide dismutase, while had no significant effect on inflammatory factors. Ultra-performance liquid chromatography/quadrupole-time-of-flight mass spectrometry (UPLC/Q-TOF-MS) analysis detected 30 prototype components in model rats' serum, mainly including alkaloids, saponins, terpenoids, tanshinones, phenols. UPLC-MS/MS successfully detected the pharmacokinetic parameters of four components, and correlation analysis shows that there are negative correlations between four compounds and serum NT-proBNP. Thirty components of ESZWD may play a therapeutic role in chronic heart failure with heart-kidney Yang deficiency (CHF-HKYd) by improving myocardial injury, reducing oxidative stress levels, and inhibiting activation of the RAAS system in rats. Salsolinol, aconitine, paeoniflorin, and miltrione are equipped with potential characteristics as pharmacodynamic substances for ESZWD in treating CHF-HKYd. Additionally, the constituents of ESZWD in CHF-HKYd rats are different from normal rats, which provided a reference for the selection of subjects for further study.

## Introduction

Chronic heart failure (CHF) is a complex clinical syndrome of ventricular ejection and/or filling caused by a variety of cardiac structural or functional diseases, it is the end-stage manifestation of cardiovascular diseases and the main cause of death. According to the epidemiological data in recent years, there are about 4.5 million patients with heart failure (HF) in China, accompanied by gender and regional differences (1, 2). In modern times, as a dual inhibitor of angiotensin receptor and enkephalin enzyme, shakubactrivalsartan has been listed in the international guidelines for HF, and its efficacy is remarkable. Regrettably, there are still some adverse reactions to shakubactrivalsartan, such as angioedema, hypotension, renal function impairment, hyperkalemia, and so on. Therefore, looking for a more appropriate therapeutic paradigm is currently a concern to be solved in CHF treatment. Under this situation, people gradually turn their attention to Traditional Chinese Medicine (TCM), which can greatly ameliorate the life quality and the medication compliance of patients as well as decrease the side effects compared with western medicine, they therefore have a good application prospect.

Ershen Zhenwu Decoction (ESZWD) is a classical prescription of Xin'an Cheng's internal medicine in treating CHF, which is composed of *Aconiti* Lateralis Radix Praeparata, *Poria, Atractylodis Macrocephalae* Rhizoma, *Paeoniae* Radix Alba, *Zingiberis Rhizoma* Recens, *Ginseng* Radix et Rhizoma Rubra and *Salviae Miltiorrhizae* Radix et Rhizoma, clinical records show that it has significant effects in the treatment of chronic heart failure with heart-kidney Yang deficiency (CHF-HKYd) (3–14). Despite the definite effects of ESZWD on CHF-HKYd, these findings still leave open the question that what are the effective material substances of this prescription. Due to the complex composition of TCM compounds, their efficacy has always been explained by TCM theories, in order to cope with this issue, the focus of modern TCM research is to explore the pharmacodynamic substances and mechanisms (15). Here, we refer to the idea of serum spectroscopic study, first, the chromatographic technique is used to characterize the chemical information of the serum components of TCM, and then the pharmacological experiments are combined to obtain the efficacy information of TCM, finally, the “spectrum-effect relationship” of TCM is constructed through relevant analysis methods. This research model remedy for the drawbacks of traditional spectrum effect research that the filtration and metabolism of TCM in the body are not taken into consideration (16), promotes the research of effective substances of TCM.

In the previous research of our group, the ultra-performance liquid chromatography/quadrupole time-of-flight mass spectrometry (UPLC/Q-TOF-MS) technology has been used to analyze the chemical components of ESZWD and its serum components in normal rats (17). In this present study, we set up to create the CHF-HKYd rat model by bilateral thyroidectomy combined with doxorubicin injection and explore the effects of ESZWD on model rats. Then, the chemical constituents profiling of ESZWD in CHF-HKYd rats serum was carried out by utilizing UPLC/Q-TOF-MS technology and screened out for representative chemical components. Finally, the pharmacokinetic parameters of the four chemical components in the model rats and the changes of serum N-terminal pro-brain natriuretic peptide (NT-proBNP) levels at different time points were determined by UPLC-MS/MS and ELISA kit, respectively, and the correlation analysis method was used to investigate the relationship between four components and NT-proBNP level. This study is in order to provide references for pharmacodynamic substances exploration of ESZWD.

## Instruments and material

Doppler electrocardiograph (CHISON Medical Technologies Co., Ltd, China), Multiskan Spectrum Full Wavelength Microplate (Thermo Fisher Scientific, USA), Waters ACQUITY-CSH-C_18_ column, Waters ACQUITY^TM^ ultra performance liquid chromatography, Waters XEVO G2 Q-TOF high resolution time-of-flight mass spectrometer (Waters, USA), IKA RV10 digital rotary evaporator (IKA, Germany), LC-4016 low speed centrifuge (CZJ Scientific Instrument Co., Ltd.), Milli-Q Gradient A10 Ultra-Pure Water System [Milliple (Shanghai) Trading Co., Ltd.], Micropipette gun (Eppendorf, Germany).

Paeoniflorin (LOT: 110736201539) was purchased from the State Food and Drug Administration; Aconitine (LOT: 15121111), ginsenoside Rb1 (LOT: Z20S9X70603), miltrione (LOT: W29A10Z96532), Salsolinol (LOT: A22GB146211) were purchased from Shanghai Yuanye Biotechnology Co., Ltd., TanshinoneIIA (LOT: DST191017-011) was purchased from Chengdu Deste Biotechnology Co., Ltd. Paeoniflorin (No. ZLSW201128-1) was purchased from Xi'an Chengxiang Biotechnology Co., Ltd.

*Aconiti* Lateralis Radix Praeparata (Aconiti) was purchased from Jiangsu Huahong Pharmaceutical Technology Co., Ltd., No. 181027; *Poria* (Poria) was purchased from Anhui Linlan Co., Ltd., No. 1901001; *Atractylodis Macrocephalae* Rhizoma (Atractylodes, Atractylodis Macrocephalae) was purchased from Anhui Hua Tianbao Traditional Chinese Medicine Decoction Pieces Co., Ltd., No. 1901001; *Paeoniae* Radix Alba (Paeonia L., Paeoniae) was purchased from Bozhou Chengyuan Traditional Chinese Medicine Pieces Co., Ltd. No. 20181101; *Salviae Miltiorrhizae* Radix et Rhizoma (Salvia Linn., Salviae Miltiorrhizae) was collected from Taihe County, Anhui Province. *Ginseng* Radix et Rhizoma Rubra (Panax L., Ginseng) was purchased from Zhejiang Zhenyuantang TCM Decoction Pieces Co., Ltd., No. 20191001; *Zingiberis* Rhizoma Recens (Zingiber, Zingiberis) is homemade. All the medicinal materials were identified by Professor Yu Nianjun of Anhui University of Traditional Chinese Medicine and met the standards of the Chinese Pharmacopoeia (2020 edition).

## Experimental animals

All male rats were kept at a room temperature of 20 ± 2°C with a 12-h light/dark cycle and 50 ± 5% relative humidity for 1 week. Animals reported here have been approved by the Institutional Animal Care and Use Committee of Anhui Medical University [Certification of fitness number: scxk (Anhui) 2017-001], and the experimental procedures were conducted in accordance with the Guidelines for Proper Conduct of Animal Experiments. Animal experiments have been approved by the Ethics Committee of Anhui University of Chinese Medicine (Ethics Number: AHUCM-rats-2021032).

## Methods

### Replication and evaluation of CHF-HKYd rat model

SPF SD male rats were anesthetized by intraperitoneal injection of 25% urethane (1,000 mg/kg). Cut off the skin tissue for about 1 cm, blunt away from bilateral neck muscles, separate and excise the bilateral thyroid glands, and the parathyroid glands were retained. The mixture of noradrenaline and normal saline (1:4,000) was used to stop intraoperative bleeding during the operation, the muscle and skin were sutured and 0.1% calcium gluconate solution was given postoperatively. On the 10th day after thyroidectomy, the rats were intraperitoneally injected with 0.02% doxorubicin hydrochloride (1 mL/100 g), twice a week for 3 weeks.

The general state of rats was observed: the basic criteria for the symptoms of HKYd were dark purple coating on the tongue, asthma, edema, fear of cold, emaciation, loose stools, sleepiness, hypotrichosis, etc. The urine volume of rats was measured: 1 week after the last injection, 24 h urine volume of rats in the normal and model group were collected in the metabolic cage, and the urine samples were frozen at −80°C. Evaluation of cardiac function in rats by Doppler echocardiography: After urine collection, the rats were anesthetized and fixed in the supine position. The precardiac area of the rats was depilated and the coupling agent was applied, a frequency of 12 MHz. M-mode ultrasound and pulsed Doppler ultrasound were used to detect diastolic interventricular septum (IVSD), left ventricular internal diastolic diameter (LVIDd), left ventricular posterior wall diastolic diameter (LVPWd), systolic interventricular septum (IVSs) and left ventricular internal systolic diameter (LVIDs), left ventricular posterior wall systolic diameter (LVPWs), stroke volume (SV), left ventricular ejection fraction (LVEF), left ventricular short-axis systolic fraction (FS), and the ratio of peak flow velocity between early and late mitral valve diastole (E/A) through the left ventricular macropinacoid of left sternal bone and four-chamber view. Neuroendocrine factor detection: After the echocardiography, 1 mL of blood was taken from the canthus of rats in each group, blood was placed in the water at 37°C for 1.5 h, centrifuged to obtain serum (3,000 rpm, 10 min), frozen at −80°C. The contents of serum NT-proBNP, T_3_, and T_4_ were detected by ELISA.

### Preparation of ESZWD solution

*Aconiti* Lateralis Radix Praeparata soaked (3.0 g) in 10 times distilled water decocted for 0.5 and 1 h with high and low temperature, respectively. Afterward, they were decocted with other six herbs including *Ginseng* Radix et Rhizoma Rubra (6.0 g), *Poria* (10.0 g), *Atractylodis Macrocephalae* Rhizoma (10.0 g), *Paeoniae* Radix Alba (10.0 g), *Salviae Miltiorrhizae* Radix et Rhizoma (30.0 g), *Zingiberis* Rhizoma Recens (6.0 g) for 0.5 h (18), filtered, and the residue decocted with eight times water for 0.5 h again. Finally, the two filtrates were combined and concentrated under reduced pressure in the rotary evaporator for centrifugation, the supernatant was vacuum dried and stored at −20°C after vacuum drying. The accurately weighed 10.0 g freeze-dried powder was dispersed in 1 mL initial proportion of mobile phase, then centrifuged at high speed (12,000 rpm·min^−1^, 10 min), filtered through 0.22 μm membrane, and analyzed.

### Animal treatment and index detection

All rats were randomly divided into control group, model group, ESZWD low-dose (3.96 g/kg), medium-dose (7.92 g/kg), high-dose (15.84 g/kg) groups, and positive control group (benazepril, 0.5 mg/kg), with six rats in each group. The CHF-HKYd was established after 1 week of feeding. ESZWD and benazepril were administered by gavage separately for 4 weeks after successful modeling, except for the control group. Blood was collected for 5, 10, 20, 30, 45 min, 1, 2, 4, 6, 8, 12, and 24 h, standing for 2 h and centrifuged at 4°C, 3,500 rpm for 10 min, the supernatant was retained. The state of rats was observed and the urine volume at 24 h was measured. The structure and function of rat heart were evaluated by doppler ultrasonography. Serum biomarkers including myocardial injury indicators NT-proBNP, angiotensinII (AngII), aldosterone (ALD), reactive oxygen species (ROS), superoxide dismutase (SOD), malondialdehyde (MDA), and tumor necrosis factor α (TNF-α) and interleukin 6 (IL-6) were detected by ELISA.

### Test conditions of ESZWD in serum

#### Chromatographic conditions

Chromatographic analysis was performed using ACQUITY UPLC BEH C_18_ column (2.1 × 50 mm, 1.7 m), the mobile phase was acetonitrile (A) 0.1% formic acid water (B), the flow rate was set as 0.2 mL·min^−1^, the column temperature was 35°C, the injection volume was 2 μL, and the gradient conditions were as follows: 0–8 min: 6–11%A, 8–23 min: 11–19%A, 23–45 min: 19–38%A, 45–55 min: 38–47%A, 55–55.1 min: 47–52%A, 55.1–65 min: 52–90%A, 65–66 min: 90–6%A of UPLC-Q-TOF/MS analysis and 0–3 min: 8–10%A, 3–3.5 min: 10–70% A, 3.5–5: 70–80%A, 5–5.1 min: 80–8% A, 5.1–6 min: 8% A of UPLC-MS/MS.

#### Mass spectrometry conditions

Mass spectrometric detection of UPLC-Q-TOF/MS was carried out on electrospray ionization (ESI) ion source, the scanning range of *m/z* 50–1,200 Da, scan time is 0.5 S, capillary voltage is 3.0 kV in positive mode and 2.5 kV in negative mode, cone voltage is 40 V, source temperature is 120°C (ESI^+^) and 110°C (ESI^−^), desolvation gas temperature of 350°C, desolvation gas (N_2_) flow of 600 L·h^−1^, collision energy is 35 V in positive mode and −10 V in negative mode, vertebral hole gas velocity: 50 L·h^−1^, the quality of the correction fluid for Leu-enkephalin, nucleo ratio of *m/z* 556.2766 in positive mode and *m/z* 554.2620 in negative mode.

Mass spectrometric detection of UPLC-MS/MS analysis was carried out on electrospray ionization (ESI) ion source, multiple reaction monitoring (MRM) mode, ion spray voltage of 5,500 V, the curtain gas and collision gas were 40 and 9 kPa, respectively, declustering potential (DP) and collision voltage (CE) are shown in Table 1.

**Table 1:** Mass spectral parameters of five compounds.

**Compound**	**m/z (Da)**	**DP (V)**	**CE (V)**
Salsolinol	179.4/145.0	69.04	26.90
Aconitine	645.5/586.4	111.98	50.88
Miltrione	284.2/88.0	107.29	31.83
Paeoniflorin	480.3/121.9	10.99	30.94
TanshinoneIIA	302.2/88.1	118.93	47.06

### Data processing and analysis

For cytokine testing: Results were expressed as mean ± standard deviation ($\bar{x}$ ± SD), SPSS 25.0 software was used for statistical analyses. The Kruskal–Wallis test, one-way analysis of variance (ANOVA), and the least-significant difference (LSD) test were used for statistical parameter analysis. *P* < 0.05 was regarded as statistically significant.

For chemical composition analysis: By searching online databases (CNKI, PubMed and WebScience, and PubChem, ChemBook, and ChemSpider), the ESZWD chemical composition database was constructed and imported into UNIFI software. Using the UNIFI^TM^ data analysis platform to identify the chromatographic peaks of ESZWD whole formula and disassembled formula; A binary sample model was used for comparative analysis, that is, between the blank solvent sample and the sample solution. Besides, in the positive ion mode, [M+H]^+^ or [M+Na]^+^ were selected as the main additive ion mode, and in the negative ion mode, [M-H]^−^ or [M+HCOO]^−^ were selected as the main additive ion mode, and the upper and lower limits of molecular weight deviation of ESZWD and its disassembled formula were set as 10 ppm.

## Results

### Evaluation of CHF-HKYd rat model

#### Doppler echocardiography

**Figure 1:**
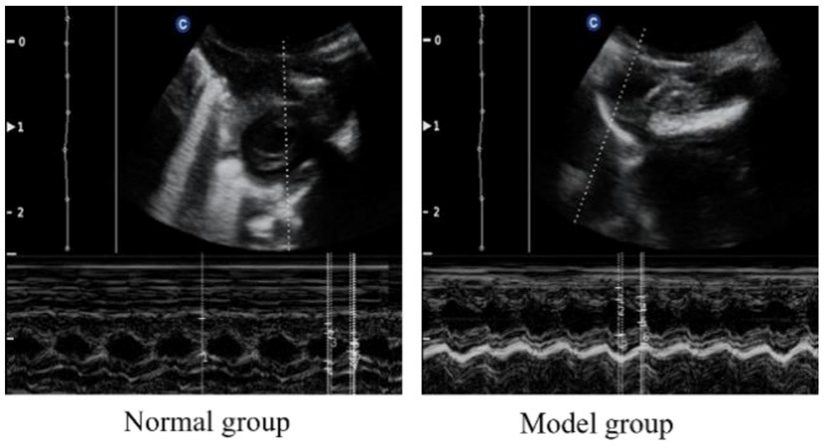
Doppler echocardiography of normal and CHF-HKYd rats.

To verify the success of the model, we examined the cardiac function of the rats. The cardiac function of each rat was analyzed by echocardiography after modeling. As can be seen from Figure 1 and Table 2, compared with the normal group, the IVSD (mm) and LVEDD (mm) in the model group were significantly increased, the E/A < 1. This indicated that the diastolic function of the model rats was impaired.

**Table 2:** Doppler cardiac function parameters of normal and CHF-HKYd rats.

	**IVSd/mm**	**LVIDd/mm**	**LVPWd/mm**	**IVSs/mm**	**LVIDs/mm**
Normal group	1.180 ± 0.11	4.480 ± 0.50	1.260 ± 0.23	2.140 ± 0.79	1.140 ± 0.40
Model group	1.460 ± 0.19^#^	5.820 ± 0.86^#^	1.420 ± 0.40	2.380 ± 0.66	1.760 ± 0.72
	**SV/mL**	**LVEF%**	**FS%**	**LVPWs/mm**	**E/A**
Normal group	0.454 ± 0.47	97.55 ± 2.62	74.25 ± 8.38	3.220 ± 0.80	2.399 ± 0.24
Model group	0.456 ± 0.21	95.35 ± 5.70	69.41 ± 12.52	2.500 ± 1.08	0.756 ± 0.08^#^

Compared with normal group,

^#^P < 0.05, difference has significance.

#### Serum NT-ProBNP, T_3_, T_4_ level in normal and model rats

**Figure 2:**
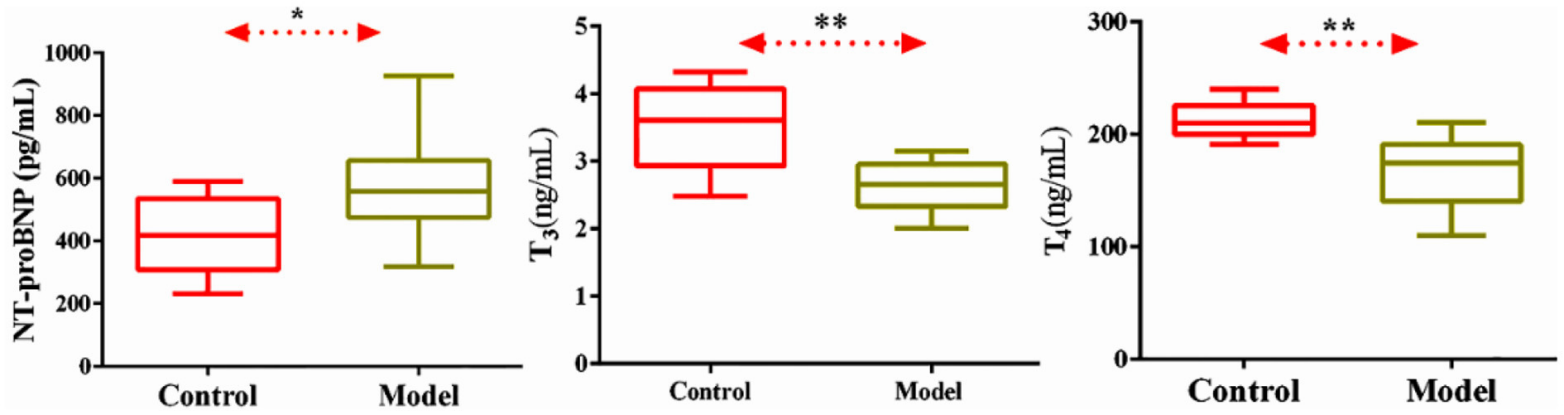
Level of serum NT-proBNP, T_3_, T_4_ in normal and CHF-HKYd rats. Compared with control group, *P < 0.05. **P < 0.01.

In order to observe the level of neuroendocrine factors changes in the model rats, we detected NT-proBNP, T_3_, and T_4_. In Figure 2, compared with the normal group, the serum levels of NT-proBNP, T_3_, and T_4_ in the model group were significantly decreased (*P* < 0.05). NT-proBNP is one of the specific markers of CHF, increased levels of this factor indicate the occurrence of CHF, while decreased levels of T_3_ and T_4_ indicate thyroid insufficiency.

### Effects of ESZWD on CHF-HKYd rats

#### Effects of ESZWD on cardiac function in CHF-HKYd rats

In Table 3, IVSD, LVED, and LVIDS of model rats were significantly increased in comparison with normal rats, while LVEF, FS, and E/A were significantly decreased, indicating decreased diastolic function and ejection ability in response to CHF-HKYd rats. Low and medium doses of ESZWD could significantly reduce LVIDd, while LVIDs was reduced only in the medium-dose group, all doses could significantly improve the E/A ratio.

**Table 3:** Effects of ESZWD on cardiac function of CHF-HKYd rats.

	**IVSd/mm**	**LVIDd/mm**	**LVPWd/mm**	**IVSs/mm**	**LVIDs/mm**	**SV/mL**	**LVEF%**	**FS%**	**LVPWs/mm**	**E/A**
Normal	1.213 ± 0.29	5.288 ± 0.52	1.650 ± 0.37	2.538 ± 0.86	2.363 ± 0.81	0.3138 ± 0.09	88.64 ± 9.03	57.53 ± 15.98	1.685 ± 0.25	1.542 ± 0.15
Model	1.643 ± 0.27^#^	6.129 ± 0.33^#^	1.671 ± 0.22	2.243 ± 0.38	3.757 ± 0.70^##^	0.3929 ± 0.07	73.55 ± 12.7^##^	38.73 ± 9.89^##^	1.891 ± 0.10	0.729 ± 0.12^#^
ESZWD-L	2.033 ± 0.58	5.633 ± 0.25*	2.000 ± 0.36	3.067 ± 0.25	3.433 ± 0.67	0.3100 ± 0.09*	76.99 ± 16.0	38.65 ± 13.4	2.667 ± 0.45	1.863 ± 0.43*
ESZWD-M	1.817 ± 0.57	5.450 ± 0.63*	2.017 ± 0.38	2.767 ± 0.30	2.900 ± 0.87*	0.3183 ± 0.08	82.41 ± 9.61	47.66 ± 12.2	3.033 ± 0.66**	2.013 ± 0.42*
ESZWD-H	1.433 ± 0.12	6.133 ± 0.80	1.733 ± 0.35	2.367 ± 0.23	3.60 ± 0.56	0.4767 ± 0.22	84.37 ± 8.85	49.65 ± 12.4	2.933 ± 0.68*	1.976 ± 0.36*
Benazepril	1.800 ± 0.62	5.950 ± 1.28	1.900 ± 0.67	3.425 ± 0.88	2.350 ± 0.72**	0.4950 ± 0.25	92.30 ± 4.09**	60.31 ± 8.10**	3.525 ± 0.79**	1.794 ± 0.28*

Compared with control group,

^#^P < 0.05,

^##^P < 0.01, difference has significance; Compared with model group,

^*^P < 0.05,

^**^P < 0.01, difference has significance.

### Effects of ESZWD on serum levels of NT-proBNP, ALD, and AngII in CHF-HKYd rats

**Figure 3:**
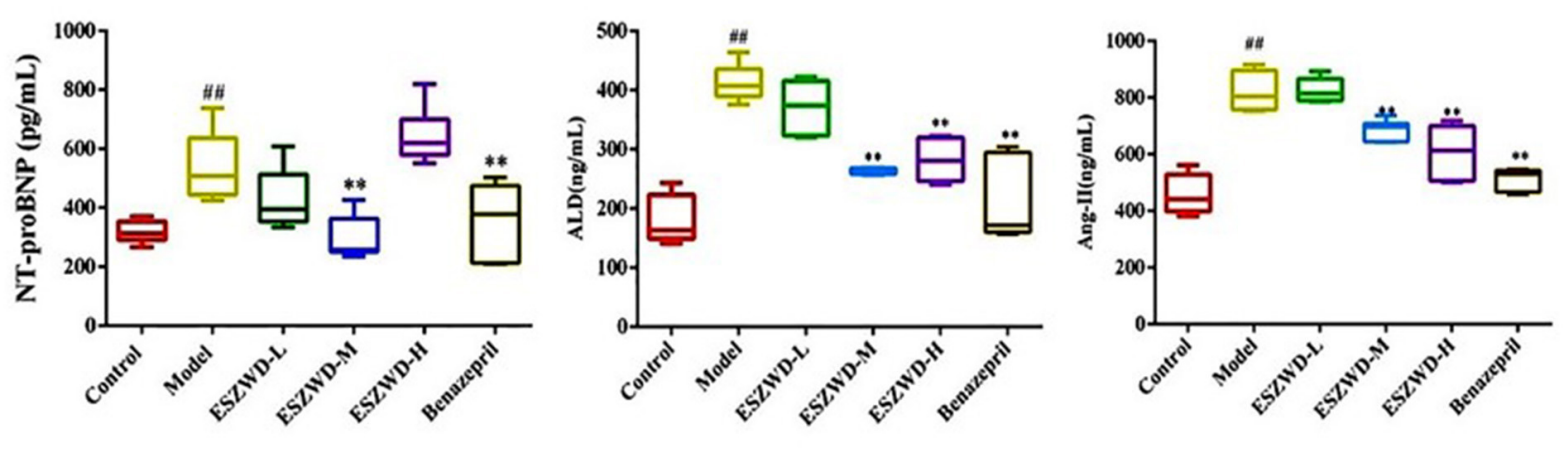
Effects of ESZWD on serum NT-proBNP, ALD, and AngII level of CHF-HKYd rats. Compared with control group, ^##^*P* < 0.05; compared with model group, **P < 0.01.

As can be seen from Figure 3, compared with the normal group, serum NT-proBNP, ALD and AngII were significantly increased in the model group. Compared with the model group, medium and high doses of ESZWD could significantly reduce the level of NT-proBNP, and medium and high doses of ESZWD could significantly reduce the levels of ALD and AngII in serum. Benazepril significantly reduced the contents of these three cytokines.

### Effects of ESZWD on serum levels of ROS, MDA, and SOD in CHF-HKYd rats

**Figure 4:**
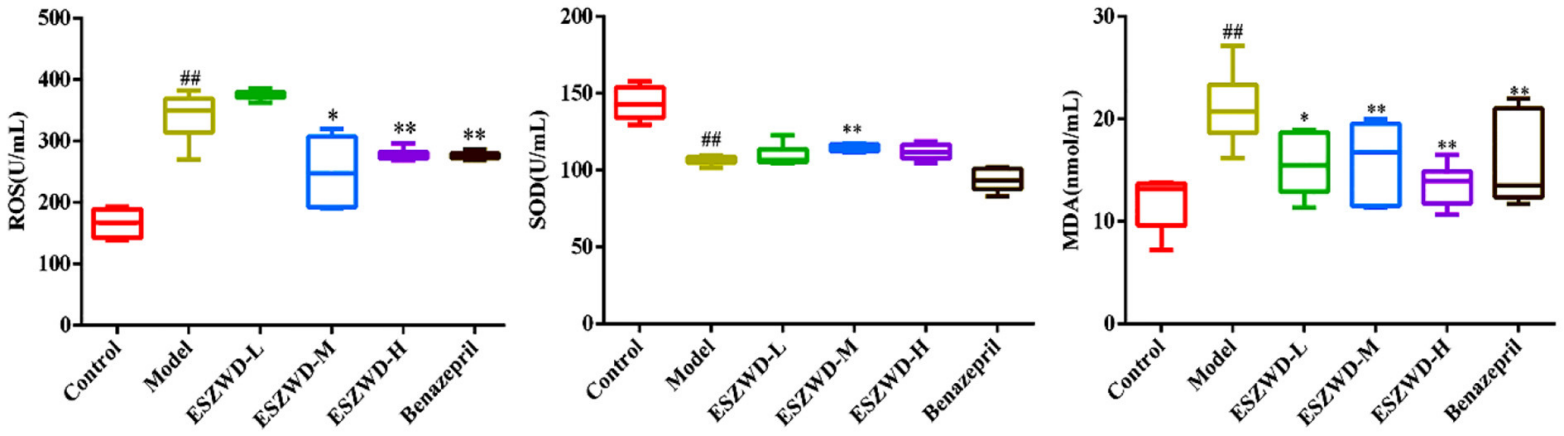
Effects of ESZWD on serum ROS, SOD, and MDA level of CHF-HKYd rats. Compared with control group, ^##^*P* < 0.05; compared with model group, *P < 0.05, **P < 0.01.

As illustrated in Figure 4, compared with the normal group, serum ROS and MDA in the model group were significantly increased, while SOD was significantly decreased. Obviously, medium and high doses of ESZWD could significantly reduce ROS and MDA levels, while SOD was increased only in ESZWD medium-dose group. Additionally, benazepril can significantly reduce ROS and MDA levels.

### Effects of ESZWD on serum levels of TNF-α and IL-6 in CHF-HKYd rats

**Figure 5:**
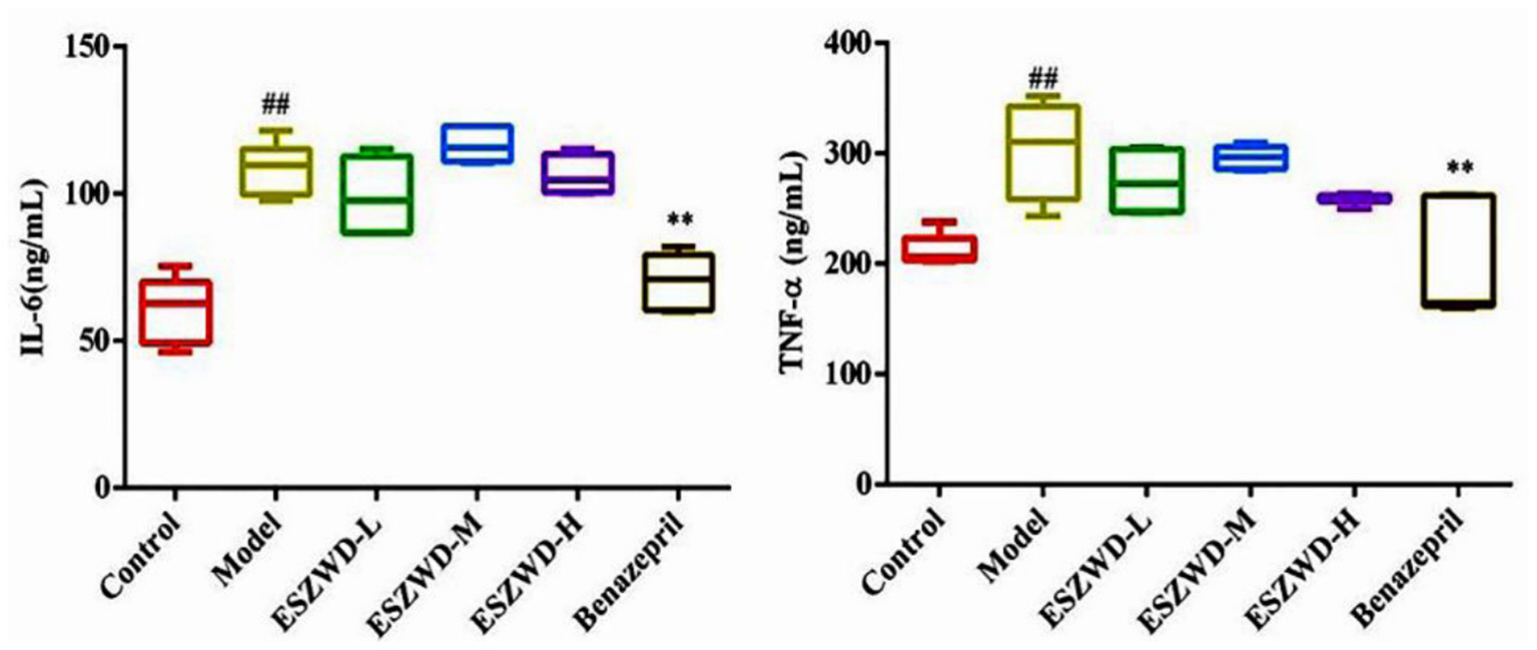
Effects of ESZWD on serum TNF-α and IL-6 level of CHF-HKYd rats. Compared with control group, ^##^*P* < 0.05; compared with model group, **P <0.01.

As shown in Figure 5, compared with the normal group, serum TNF-α and IL-6 in the model group were significantly increased, indicating that inflammation occurred in the model group. Compared with the model group, ESZWD had no significant effects on TNF-α and IL-6, indicating that these two cytokines may not be the therapeutic targets of ESZWD, but it is not excluded that ESZWD may inhibit inflammation. Benazepril significantly reduced levels of both inflammatory factors.

### Chemical compositions of ESZWD in CHF-HKYd rats

#### Analysis of main components of ESZWD in CHF-HKYd rats

As shown in Table 4, there were 30 compounds of ESZWD identified in CHF-HKYd rats, including alkaloids, saponins, 3 terpenoids, tanshinone, phenols, individual fat, fatty acids, flavonoids, and sulfonate, nucleic acid compounds. There were 12 compounds from monarch medicine, 12 compounds from minister medicine, and 10 kinds of assistant medicine.

**Table 4:** Serum components of ESZWD in CHF-HKYd rats.

**Peak**	**Component name**	**Formula**	**Observed RT (min)**	**Observed m/z**	**Fragment ions (m/z) (±)**	**Mass error (ppm)**	**Origin**
1	Salsolinol	C_10_H_13_NO_2_	7.22	[M+H]^+^:180.1026	146.06105	3.6	A
2	Paeoniflorin*	C_23_H_28_O_11_	8.39	[M+HCOO]^−^:525.1578	/	−6.8	BC
3	Dimethyl D-malate	C_6_H_10_O_5_	11.41	[M+H]^+^:163.0597	133.01354	−2.4	B
4	letestuianin B	C_21_H_22_O_6_	13.86	[M+H]^+^:371.1515	217.08122	6.9	C
5	1-(4-Hydroxy-3-methoxyphenyl)-3-oxo-5-decanesulfonic acid	C_17_H_26_O_6_S	14.86	[M+H]^+^:359.1542	203.10301,217.08327	5.4	C
6	(1a,3a,6a,14a,15a,16b)-20-Ethyl-1,6,16-trimethoxy-4-(methoxymethyl)aconitane-3,8,13,14,15-pentol 8-Acetate 14-(4-Methoxybenzoate)	C_35_H_49N_O_12_	17.65	[M+Na]^+^:698.3217	375.14910,458.18640, 517.27153,588.30833	10	A
7	24-methylenecycloartanol	C_31_H_52_O	17.71	[M+H]^+^:441.4101	175.11843	2.3	C
8	Aconitine	C_34_H_47_NO_11_	19.69	[M+Na]^+^:341.1	173.12716,201.12205,479.21224	0.1	A
9	Acetylaconitine	C_36_H_49_NO_12_	21.32	[M+Na]^+^:710.3082	175.1174,301.11698, 517.30894,588.30894	−9.2	A
10	(6S)-6,7,8,9-Tetrahydro-6-hydroxy-6-hydroxymethyl-1-methylphenanthro[1,2-b]furan-10,11-dione	C_18_H_16_O_5_	24.11	[M+HCOO]^−^:357.0952	225.12635	−7.7	B
11	Adenosine	C_10_H_13_N_5_O_4_	33.04	[M+H]^+^:268.1039	165.06844	−0.6	B
12	20-Ethyl-8-hydroxy-1,16-dimethoxy-4-(methoxymethyl)aconitan-14-yl acetate	C_26_H_41_NO_6_	33.73	[M+H]^+^:464.2993	209.1264,335.23656,353.25368	−2.9	A
13	Ginsenoside Rb1*	C_54_H_92_O_23_	37.63	[M+HCOO]^−^:1153.5997	/	−1.2	BC
14	Ginsenoside Rb3	C_54_H_92_O_23_	37.66	[M+HCOO]^−^:1153.5997	783.48919,945.54043	−0.2	B
15	TanshinoneIIB	C_19_H_18_O_4_	41.28	[M+H]^+^:311.1251	199.07924,213.09088	−8.6	B
16	3-Hydroxy-2-isopropyl-8-methyl-1,4-phenanthrenedione	C_18_H_16_O_3_	44.65	[M+HCOO]^−^:325.1068	265.08506	−4.3	A
17	(1α,5ξ,6β,9ξ,10ξ,13ξ,14α,16β,17ξ)-20-Ethyl-14,16-dimethoxy-4-(methoxymethyl)aconitane-1,6,7,8-tetrol	C_24_H_9_NO_7_	48.58	[M+Na]^+^:476.2603	119.08554	−3.3	A
18	(1R,4aR,7R,8aR)-7-(2-Hydroxy-2-propanyl)-1,4a-dimethyldecahydro-1-naphthalenol	C_15_H_28_O_2_	51.63	[M+Na]^+^:263.1989	95.08448	3	C
19	Poricoic acid E	C_30_H_44_O_6_	55.11	[M-H]^−^:499.3033	301.21485	−6.5	B
20	(16β,17ξ)-20-Ethyl-8,13-dihydroxy-1,16-dimethoxyaconitan-14-yl benzoate	C_29_H_39_NO_6_	56.71	[M+Na]^+^:520.2662	184.07261,319.19338	−1.5	A
21	Ginsenoside Rs1	C_55_H_92_O_23_	57.27	[M+Na]^+^:1143.591	184.06991,520.33039,544.32997	−1	A
22	Miltrione	C_19_H_22_O_2_	57.96	[M+Na]^+^:305.1516	155.00831	1.4	B
23	Ginsenoside Rk2	C_36_H_60_O_7_	58.55	[M+H]^+^:605.4366	105.06872,337.26457,426.35458	−7.5	A
24	(16β)-3,13,15-Trihydroxy-1,6,8,16-tetramethoxy-4-(methoxymethyl)-20-methylaconitan-14-yl benzoate	C_31_H_57_NO_10_	60.97	[M+Na]^+^:626.2949	104.10528,184.06999	2.1	A
25	3-Phenanthrenol, 4b,5,6,7,8,8a,9,10-octahydro-4b,8,8-trimethyl-2-(1-methylethyl)-, (4bS,8aS)-	C_20_H_30_O	63.12	[M+H]^+^:287.2375	95.08582,121.10144	1.9	B
26	Lecithin	C_42_H_81_NO_8_P^+^	64.78	[M+H]^+^:758.5661	104.10657,187.01850,362.18849	−4.3	C
27	Ginsenoside Rk3	C_36_H_60_O_8_	65.12	[M+HCOO]^−^:665.4253	281.24682	−2.6	ABC
28	2-Methyl-5-[(2R)-6-methyl-5-hepten-2-yl]phenol	C_15_H_22_O	65.28	[M+Na]^+^:241.1546	95.08448	−6.8	C
29	kaempferol 3,7-di-O-β-D-glucoside	C_27_H_29_O_16_	66.17	[M+HCOO]^−^:655.1566	223.02565	−6.3	B
30	1,2,3,4,6-Pentagalloyl glucose	C_41_H_32_O_26_	66.3	[M+Na]^+^:963.1134	355.0616	6.2	C

^*^Contrast with reference solution.

A, Monarch medicine; B, Minister medicine; C, Assist medicine.

### Establishment of UPLC-MS/MS for simultaneous determination of four chemical components in ESZWD

#### Validation of method

##### Specificity and linear ranges

The specificity results (Supplementary Figure 1) indicated that endogenous substances did not substantially interfere with the retention time of probe drugs and internal standard (IS) in blank serum. The linear ranges of the four compounds were 10.00–1000.00, 0.40–200.00, 0.40–200.00, 0.50–200.00 ng/mL and the correction coefficients (r) were 0.9984, 0,9981, 0.9993, 0.9993, respectively.

##### Precision and accuracy

Interday precision and intraday precision of the method were assessed by detecting the low limit of qualification (LLOQ) and the low-, medium-, and high-quantification concentrations (LQC, MQC, and HQC) of plasma samples. Relative standard deviation (RSD) values of the precision do not exceed ±15% (Supplementary Tables 1, 2).

##### Matrix effect

By comparing the different results of analytes added into the blank sample and ultrapure water, the matrix effect was determined at LQC, MQC, and HQC concentrations. The RSD values (Supplementary Table 3) were <4%, indicating that the matrix effect of plasma is negligible for quantitative analysis of all samples.

##### Stability

The stability of all probe drugs was evaluated by LQC and HQC samples under different experimental conditions, including short-term stability [4 h at room temperature (25°C), 8 h in the automatic sampler, and three freeze and thaw cycles, respectively] and long-term stability (7 d at −80°C). Results showed that the probe substrates tested were within the recommended limits, RSD < 10% (Supplementary Table 4).

#### Pharmacodynamic substances of ESZWD in treating CHF-HKYd

##### Pharmacokinetic parameters of four chemical components in ESZWD in CHF-HKYd rats

**Figure 6:**
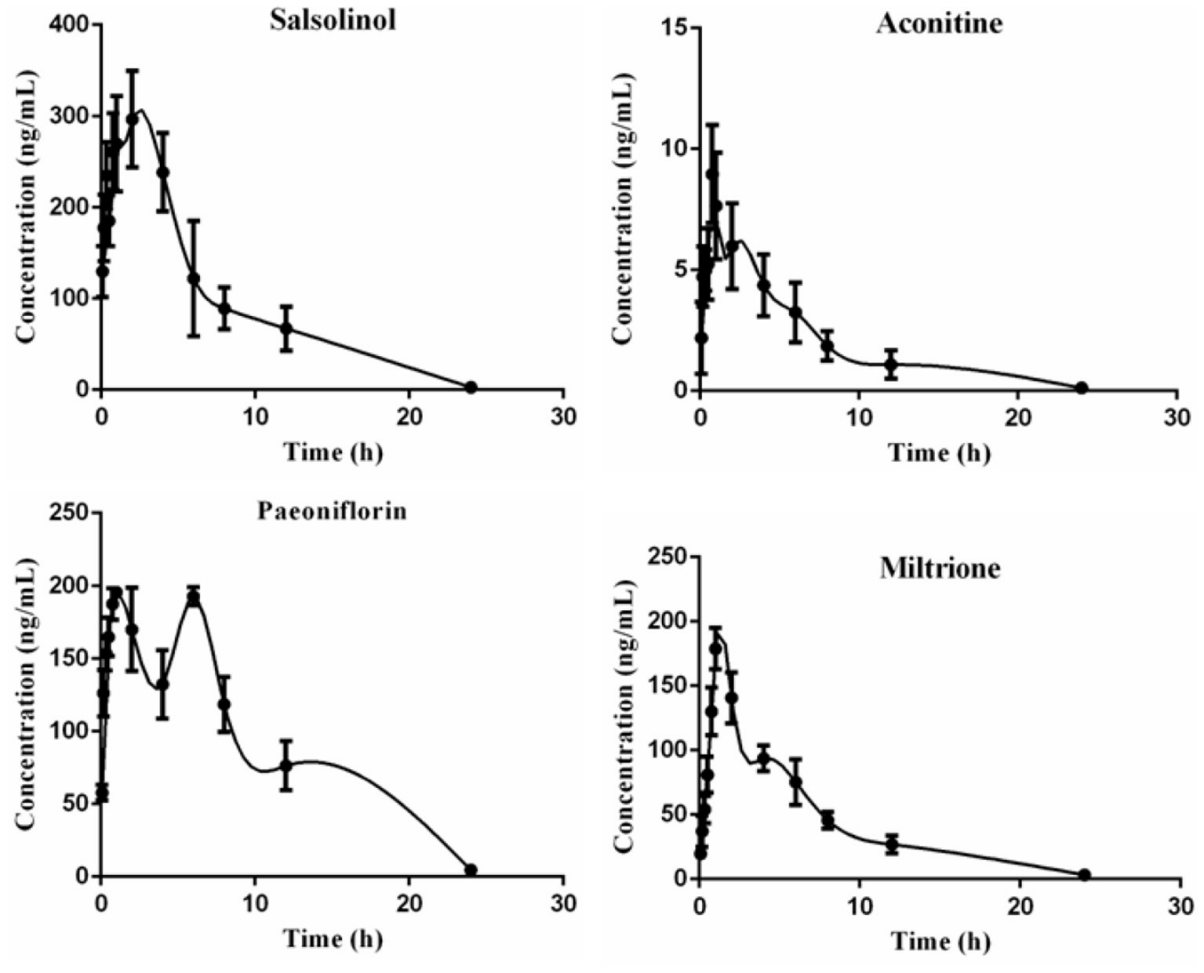
Time-concentration curves of salsolinol, aconitine, paeoniflorin and miltrione in CHF-HKYd rat serum ($\bar{x}$ ± SD, *n* = 6).

Results are presented in Table 5 and Figure 6, and the area under the curve (AUC) indicates the amount of drug exposure in the body, volume (V) is positively correlated with clearance (CL) and negatively correlated with C_max_. The content of salsolinol in the body is large, half-life time (T_1/2_) is about 3 h, CL is low, V is small, T_max_ is 1.25 h, indicating it is a compound with fast absorption and elimination. With the bimodal phenomenon, paeoniflorin is higher in the serum, T_1/2_ is about 3 h, CL is low, V is small, and T_max_ is 6 h. Aconitine has the lowest AUC, supporting that it is present at very low levels in the body, which may be the reason that the compound is effective but not toxic, given its highly toxic nature. In addition, the T_max_ of this compound is only 0.75 h, and the T_1/2_ is about 4 h, which is a compound with fast absorption and slow elimination. The V is far more than the body fluid, it is speculated that aconitine is a fat-soluble compound and is mainly distributed in the tissues and organs rich in fat. The pharmacokinetic characteristic of miltrione is similar to salsolinol, but the AUC is much less than salsolinol while the V is higher, so miltrione enters into tissues more easily.

**Table 5:** Main pharmacokinetic parameters of salsolinol, aconitine, paeoniflorin, and miltrione in CHF-HKYd rat serum ($\bar{x}$ ± SD, *n* = 6).

	**Salsolinol**	**Aconitine**	**Paeoniflorin**	**Miltrione**
AUC_(0−t)_ (ng/mL·h)	2,327 ± 261.0	48.94 ± 16.43	2239 ± 177.8	1,098 ± 69.72
AUC_(0−∞)_ (ng/mL·h)	2,344 ± 265.7	50.11 ± 16.01	2258 ± 178.8	1,125 ± 84.73
T_1/2_ (h)	3.271 ± 0.58	4.24 ± 1.83	3.223 ± 0.34	4.25 ± 0.82
T_max_ (h)	1.25 ± 0.59	0.75 ± 0.00	6.00 ± 2.54	1.00 ± 0.00
CL/F (L/h/kg)	8.625 ± 0.96	424.7 ± 97.65	8.86 ± 5.55	17.87 ± 1.38
V/F (L/kg)	40.68 ± 9.03	2679 ± 1574	41.20 ± 0.83	108.7 ± 15.80
C_max_ (ng/mL)	314.2 ± 36.84	8.96 ± 2.22	197.6 ± 27.66	178.9 ± 16.24

#### Relationship between four compounds and serum NT-ProBNP level

Serum NT-proBNP levels at different time points are shown in Supplementary Table 4, correlation analysis results showed that salsolinol, aconitine, paeoniflorin, and miltrione were negatively correlated with serum NT-proBNP level, with correlation coefficients of −0.576 (*P* < 0.05), −0.753 (*P* < 0.05), −0.705 (*P* < 0.05), −0.477, respectively.

## Discussion

In this study, the CHF-HKYd rat model was replicated by bilateral thyroidectomy combined with adriamycin injection, and the therapeutic effects of ESZWD on the model rats were explored from the perspectives of cardiac function, myocardial injury, oxidative stress, and inflammatory response. The results showed that ESZWD could significantly reduce LVIDd and increase E/A value in model rats, medium and high doses of ESZWD could significantly reduce the levels of Ang II and ALD in serum, and NT-proBNP level was reduced only in the medium-dose group. The serum ROS and MDA levels were significantly reduced while the SOD level was increased only in the medium-dose group. However, the formula had no significant effects on the contents of TNF-α and IL-6 in serum. Echocardiography is commonly performed to evaluate cardiac diastolic function in clinical practice as a convenient and non-invasive means, and both the LVIDd and E/A values are ordinarily used indicators. NT-proBNP (19) is produced by myocardial cells vascular peptide hormones, the value of this cytokine will be significantly increased during HF and ejection fraction decreased process, leading to ventricular wall expansion and myocardial remodeling, accelerating the progression of the disease. Ang II and ALD are polypeptide substances and steroid hormones produced by RAAS system activation, one of the important indicators in ventricular remodeling. Ang II (20) has the ability to promote the ALD release by combining with ATR1 and triggering an inflammatory response, thus, inhibiting RAAS system activation has been recommended by the European Heart Pathology Association in the therapeutic strategy of CHF (21). Oxidative stress participates in the mechanisms of cardiovascular disease, and it is defined as the excessive production of live free radicals. ROS (22) is a single-electron reduction product of a class of oxygen in the body, mainly including O2∙, ∙OH, H2O2, etc, excessive ROS in the heart is involved in the processes of reduced systole function, hypertrophy signal transduction, myocardial growth, matrix remodeling, fibroblast proliferation, etc. However, the dangerous effects of ROS can be prevented by scavenging enzymes such as SOD and glutathione peroxidase (GSHPx). SOD (23) is a vital antioxidant enzyme that is able to catalyze the disproportionation of superoxide anion radicals to produce O and H2O2, it is often measured together with MDA, a final product of lipid peroxidation with the content often reflects the degree of lipid peroxidation and cell damage in the body. Studies have shown that elevated serum MDA in CHF patients increases the risk of death by 103.0% and is associated with poor prognosis (24, 25). The activation of the RAAS system is becoming more pronounced in CHF patients, and there is a positive correlation between oxidative stress and RAAS system biomarkers, for instance, Ang II is able to induce an oxidative stress response. That means to say, combined application of RAAS blockers or (and) antioxidants could be more conducive to the improvement of the disease. The experiment yielded that ESZWD has sound effects of protecting cardiac dysfunction, ameliorating oxidative stress responses including ROS and MDA levels reduction, it may be attributed to RAAS system inhibition like diminishing the production of Ang II and ALD so as to play a therapeutic role in CHF-HKYd. In the later research, the mechanism can be systematically explored in depth.

Serum components of ESZWD in normal and CHF-HKYd rats were mainly saponins, terpenoids, and alkaloids, interestingly, there were significant differences in compounds. Ginsenoside Rc, ginsenoside Rg5, ginsenoside R1, ginsenoside Rb1, ginsenoside Rg1 and ginsenoside Re were the main ginsenoside compounds in normal rats (17), while ginsenosides Rb3, ginsenosides Rs1, ginsenosides Rb1, ginsenosides Rk2 and ginsenosides Rk3 account for the main proportion in CHF-HKYd rats. Ginsenoside Rs1 can be obtained by the decarboxylation reaction of ginsenoside Rb2 (26) which is considered a new marker for distinguishing ginseng from American ginseng (27). Our previous study (28) appeared that the activities of CYP1A2, CYP2B1, CYP2C6, CYP2C11, and CYP3A1 in CHF rats were significantly reduced, as we know, CYP450 mediated 90% of drug metabolism (29), the change of metabolic enzyme activity caused by CHF-HKYd may be one of the reasons for the chemical compounds inconsistencies. The results of this study illustrated that the differences between normal and disease animals should be taken into consideration in further research on the pharmacodynamic substances of ESZWD, the animal subjects therefore should be selected reasonably.

Salsolinol (30) and aconitine are components of *Aconiti* Lateralis Radix Praeparata with strong cardiac effect, in addition, studies have reported that aconitine can significantly down-regulate cardiomyocyte hyperplasia induced by Ang II, and its toxicity can be reduced when used in combination with paeoniflorin (31–33). Paeoniflorin can protect myocardial ischemia reperfusion injury rats by increasing SOD activity, decreasing MDA level, and alleviating oxidative stress as well as apoptosis (34). Miltrione is derived from the diterpenoid quinone active ingredient in Salvia miltiorrhiza, which has the effects of inducing apoptosis of cancer cells, anti-platelet aggregation, anti-oxidation, and anti-inflammation, but its role in cardiovascular diseases protection has not been reported (35–37). Nevertheless, there is evidence that miltrione can inhibit the oxidative stress response of endothelial cells induced by LDL (38), which may be the main factor in cardiovascular disease protection and deserve further exploration. Based on UPLC-MS/MS, the pharmacokinetic behavior of these four compounds in CHF-HKYd rats was investigated. AUC indicated the compounds' exposure, it could be concluded that the absorption degree of salsolinol was the largest. According to CL results, aconitine is the most easily cleared compound in model rats. The V of aconitine, paeoniflorin, and salsolinol were larger than the total liquid content, which means the wide distribution in the body, and the tissue distribution of these ingredients are therefore should be investigated in the latter study, which may be the key critical elements affecting the efficacy of these compounds. However, Cmax lets us know that the blood concentration of aconitine was very low, as a kind of herb with strong toxicity, which may be a major cause that why aconitine exerts its efficacy but does not generate toxicity. In addition, the double peaks of paeoniflorin indicated that it was re-enriched, hepatoenteric circulation, gastric motility, uneven distribution of P-glycoprotein in the intestinal tract, and transformation of TCM components may be the causes of this phenomenon (39), while other three ingredients consistent with normal pharmacokinetic behavior. At last, correlation analysis demonstrated that these four chemicals are associated with the reduction of serum NT-proBNP, and their suitable pharmacokinetic characteristics make them have the potential to be the pharmacodynamic substances in the treatment of CHF-HKYd.

## Conclusion

In this research, we found that ESZWD process the ability to treat CHF-HKYd by reducing the levels of NT-proBNP, Ang II, ALD, ROS, and MDA in serum, and increasing the content of SOD, while having no effects on TNF-α and IL-6. The constituents of ESZWD in CHF-HKYd rats mainly include alkaloids, saponins, terpenoids, and tanshinones, among which salsolinol, aconitine, paeoniflorin, and miltrione have suitable pharmacokinetic profiles and are negatively correlated with NT-proBNP, they are equipped with potential characteristics as pharmacodynamic substances for ESZWD in treatment of CHF-HKYd. Additionally, the constituents of ESZWD in CHF-HKYd rats are different from normal rats, which provided a reference for the selection of subjects for further study.

## Data availability statement

The original contributions presented in the study are included in the article/Supplementary material, further inquiries can be directed to the corresponding author/s.

## Ethics statement

The animal study was reviewed and approved by Institutional Animal Care and Use Committee of Anhui Medical University [Certification of fitness number: scxk (Anhui) 2017-001].

## Author contributions

YZ for assisting with the sample detection and data processing of this experiment. W-dC for providing the platform for this experiment. C-yY and G-zL for their efforts in animal experiments. H-sW and X-yC for their guidance in the project design of this research. All authors contributed to the article and approved the submitted version.

## Funding

This research was supported by the Natural Science Foundation of Anhui Universities (grant number KJ2020A0380), Chinese Medicine Leading Personnel Training Object Special Project of Anhui Provincial Health, and Family Planning Commission [Chinese Medicine Development Secret (2018) grant number 23].

## Conflict of interest

The authors declare that the research was conducted in the absence of any commercial or financial relationships that could be construed as a potential conflict of interest.

## Publisher's note

All claims expressed in this article are solely those of the authors and do not necessarily represent those of their affiliated organizations, or those of the publisher, the editors and the reviewers. Any product that may be evaluated in this article, or claim that may be made by its manufacturer, is not guaranteed or endorsed by the publisher.

## Abbreviations

ALD: aldosterone
Ang II: angiotensin II
ATR1: angiotensin receptor 1
AUC: area under the curve
CE: collision energy
CHF-HKYd: chronic heart failure of heart and kidney yang deficiency
CL: clearance
CYP450: cytochrome P450
DP: declustering potential
E/A: ratio of peak flow velocity between early and late mitral valve diastole
ESI: electrospray ionization
ESZWD: er shen zhenwu Decoction
FS: left ventricular short axis systolic fraction
HOQ: high of qualification
IL-6: interleukin 6
IVSD: diastolic interventricular septum
IVSS: systolic interventricular septum
LLOQ: low limit of qualification
LOQ: low of qualification
LVEF: left ventricular ejection fraction
LVIDd: left ventricular internal diastolic diameter
LVIDs: left ventricular internal systolic diameter
LVPWd: left ventricular posterior wall diastolic diameter
LVPWs: left ventricular posterior wall systolic diameter
MDA: malondialdehyde
MOQ: medium of qualification
MRM: multiple reaction monitoring
NT-proBNP: N-terminal pro brain natriuretic peptide
RAAS: renin-angiotensin-aldosterone
ROS: reactive oxygen species
RT: retention time
SOD: superoxide dismutase
SV: stroke volume
T1/2: half-life time
TCM: traditional Chinese medicine
TIC: total ion chromatogram
TNF-α: tumor necrosis factor α
UHPLC/Q-TOF-MS: ultra-high-performance liquid chromatography/quadrupole time-of-flight mass spectrometry
V: volume.

## References

[1] Xuejuan J Jingmin Z Junbo G. Progress in heart failure epidemiology. Clin Med J China. (2013) 20:852–5.

[2] Jun H. Epidemiological characteristics and prevention strategies of heart failure in China. Chin J Heart Heart Rhythm. (2015) 3:2–3. 10.3877/cma.j.issn.2095-6568.2015.2.002

[3] Xiaoyu C Wandong Z Chonghui L Liwei Q Ruixue Z Yebin H. Effect of compound Zhenwu granules on neuroendocrine of chronic congestive heart failure patients. Chin J Inter Trad West Med Inten Criti Care. (2002) 6:317–9. 10.3321/j.issn:1008-9691.2002.06.004

[4] Yexiang Z Yebin H Huaifang Y Xiaoyu C Jian W. Controlled study measuring the effects of Wenshenyixin pill on BNP in patients with congestive heart failure. J Emerg Tradit Chin Med. (2008) 2:137–9. 10.3969/j.issn.1004-745X.2008.02.001

[5] Xiaoyu C Jing Z Lan G Gaowen H. Effect of compound Zhenwu granule on HS-CRP and BNP in patients with heart failure. Chin J Integ Med Cardio-Cerebrovascu Dis. (2009) 7:1018–9. 10.13194/j.issn.1673-842x.2017.08.016

[6] Xiaoyu C Lan G Jing Z Gaowen H. Effects of Fufangzhenwu electuary on traditional chinese medicine syndrome, plasma high sensitivity C-reactive protein and atrial natriuretic patients with chronic congestive heart failure. J Emerg Tradit Chin Med. (2010) 19:365–8. 10.3969/j.issn.1004-745X.2010.03.001

[7] Hong L Xiaoyu C Lan G. Effect of Wenshen Yixin decoction on 32 cases of chronic congestive heart failure. J Shandong Univ Trad Chin Med. (2012) 36:124–5. 10.16294/j.cnki.1007-659x.2012.02.013

[8] Huaifang Y Yebin H Xiaoyu C Jian W Wandong Z Yexiang Z. Clinical observation on the effects of Wenshenyixin pill in treating congestive heart failure. J Anhui Tradit Chin Med. (2005) 4:4–7. 10.3969/j.issn.1000-2219.2005.04.002

[9] WeiPu D Yebin H Yexiang Z Xiaoyu C Jia H. Effects of Wenshenyixin pellet on myocardial cell apoptosis in old patients with chronic heart failure. J Emerg Tradit Chin Med. (2012) 21:1053–4. 10.3969/j.issn.1004-745X.2012.07.012

[10] Koichi S Yoshihiko S Takeshi O Toshiaki O Kohji S Teruo T. Ongoing myocardial damage in chronic heart failure is related to activated tumor necrosis factor and Fas/Fas ligand system. Circ J. (2004) 68:747–50. 10.1253/circj.68.747 15277733

[11] Xiaoyu C Lan G Xiuhuan Z Huadan L Hong L. Effects of compound fufangzhenwu granule on clinical efficacy and plasma interleukin-6 and tumor necrosis factor -α in patients with chronic heart failure. Chin J Clin Healthc. (2012) 15:530–2. 10.3969/J.issn.1672-6790.2012.05.031

[12] Xiaoyu C Shuangyan Z Dan C. Effects of compound Zhenwu granule on NT-probNP and MMP-3 in patients with chronic congestive heart failure. In: Proceedings of the 2015 Annual Conference on Geriatric Medicine. Geriatric Branch of Zhejiang Medical Association. Zhejiang: Zhejiang Science and Technology Association (2015).

[13] Lan G Xiaoyu C. Effects of Zhenwu granule on TCM syndromes, McP-1 and BNP in patients with chronic heart failure. J Liaoning Unive Trad Chin Med. (2017) 19:60–3.

[14] Huaifang Y Yebin H Xiaoyu C Jian W Wandong Z Yexiang Z. Effects of Wenshen Yixin pellet on levels of inflammatory cytokines in patients with congestive heart failure. Chin J Inter Trad West Med Inten Criti Care. (2005) 3:149–52. 10.3321/j.issn:1008-9691.2005.03.006

[15] Wang M Chen DQ Chen L Liu D Zhao H Zhang ZH. Novel RAS inhibitors poricoic acid ZG and poricoic acid ZH attenuate renal fibrosis via a Wnt/β-catenin pathway and targeted phosphorylation of smad3 signaling. J Agric Food Chem. (2018) 66:1828–42. 10.1021/acs.jafc.8b00099 29383936

[16] Chunsheng Z Zhuoxi J Jiajing L Bing Z. Overview research status in serum spectrum-effect of Chinese materia medica. Chin Tradit Herbal Drugs. (2020) 51:3569–74. 10.7501/j.issn.0253-2670.2020.13.027

[17] Hong LL Zhao Y Yang CY Li GZ Wang HS Chen WD. Identification of chemical constituents *in vitro* and *in vivo* of Er Shen Zhenwu Decoction by utilizing ultra-high-performance liquid chromatography with quadrupole time-of-flight mass spectrometry. J Separ Sci. (2021) 44:4327–42. 10.1002/jssc.202100624 34665523

[18] Fu Weina Cheng X. Clinical efficacy of compound Zhenwu granule in the treatment of elderly patients with chronic heart failure. Clin J Trad Chinese Med. (2021) 33:2385–9. 10.16448/j.cjtcm.2021.1230

[19] Rørth R Jhund PS Yilmaz MB Kristensen SL Welsh P Desai AS. Comparison of BNP and NT-proBNP in patients with heart failure and reduced ejection fraction. Circ Heart Fail. (2020) 13:e006541. 10.1161/CIRCHEARTFAILURE.119.006541 32065760

[20] Špinarová M Špinar J Parenica J Špinarová L Málek F Lábr K. Prescription and dosage of RAAS inhibitors in patients with chronic heart failure in the FAR NHL registry. Vnitr Lek. (2019) 65:13–4. 10.36290/vnl.2019.004 30823832

[21] McMurray JJ Adamopoulos S Anker SD Auricchio A Böhm M Dickstein K. ESC Guidelines for the diagnosis and treatment of acute and chronic heart failure 2012: The Task Force for the Diagnosis and Treatment of Acute and Chronic Heart Failure 2012 of the European Society of Cardiology. Developed in collaboration with the Heart Failure Association (HFA) of the ESC. Eur Heart J. (2012) 33:1787–847. 10.1093/eurheartj.ehs104 22611136

[22] Mancini A Vergani E Bruno C Olivieri G Segni C Silvestrini D. Oxidative stress as a possible mechanism underlying multi-hormonal deficiency in chronic heart failure. Eur Rev Med Pharmacol Sci. (2018) 22:3936–61. 10.26355/eurrev_201806_15279 29949170

[23] Ma D Xu T Cai G Wu X Lei Z Liu X. Effects of ivabradine hydrochloride combined with trimetazidine on myocardial fibrosis in rats with chronic heart failure. Exp Ther Med. (2019) 18:1639–44. 10.3892/etm.2019.7730 31410120 PMC6676191

[24] Romuk E Wojciechowska C Jacheć W Zemła-Woszek A Momot A Buczkowska M. Malondialdehyde and uric acid as predictors of adverse outcome in patients with chronic heart failure. Oxid Med Cell Longev. (2019) 2019:9246138. 10.1155/2019/9246138 31687090 PMC6803743

[25] Reina-Couto M Afonso J Carvalho J Morgado L Ronchi FA de Oliveira Leite A. Interrelationship between renin-angiotensin-aldosterone system and oxidative stress in chronic heart failure patients with or without renal impairment. Biomed Pharmacother. (2021) 133:110938. 10.1016/j.biopha.2020.110938 33171402

[26] Xuanxuan P Xianggao L. Study on decarboxylation degradation of saponins and its products in red ginseng processing. J Jilin Agric Univ. (2000) 4:64–70. 10.13327/j.jjlau.2000.04.017

[27] Yang W Qiao X Li K Fan J Bo T Guo DA. Identification and differentiation of *Panax ginseng, Panax quinquefolium*, and *Panax notoginseng* by monitoring multiple diagnostic chemical markers. Acta Pharm Sin B. (2016) 6:568–75. 10.1016/j.apsb.2016.05.005 27818924 PMC5071635

[28] Hong LL Wang HS Cheng XY Zhang S Zhao Y Wang Q. Evaluation and clinical implication of Zhenwu decoction on seven cytochrome P450 enzyme in chronic heart failure rats. Curr Drug Metab. (2021) 22:746–55. 10.2174/1389200222666210701171047 34218778

[29] Chen JJ Zhang JX Zhang XQ Qi MJ Shi MZ Yang J. Effects of diosmetin on nine cytochrome P450 isoforms, UGTs and three drug transporters *in vitro*. Toxicol Appl Pharmacol. (2017) 334:1–7. 10.1016/j.taap.2017.08.020 28867436

[30] Anonymous. Pharmacology of Aconite. Chin Med Mod Dis Edu CN. (2012) 10:139.

[31] Ningning W Jia W Hongling T Yuguang W Yue G Zengchun M. Aconitine ameliorates cardiomyocyte hypertrophy induced by angiotensin II. Chin J Chin Mater Med. (2019) 44:1642–7. 10.19540/j.cnki.cjcmm.20190117.002 31090329

[32] Li J Zhang SH He D Wang JF Li JQ. Paeoniflorin reduced the cardiotoxicity of aconitine in h9c2 cells. J Biol Regul Homeost Agents. (2019) 33:1425–36. 10.23812/19-257A 31576730

[33] Xue Z Bingxiang Z Yuqin S Yuting Y Yanhong D Xiaofang X. Compatibility effect of aconitine and ginsenosides Rb1 on energy metabolism of primary cultured myocardial cells. Mod Tradit Chin Med Mat Med-Worl Sci Tech. (2015)17:1785–9. 10.11842/wst.2015.09.006

[34] Wu F Ye B Wu X Lin X Li Y Wu Y. Paeoniflorin on rat myocardial ischemia reperfusion injury of protection and mechanism research. Pharmacology. (2020) 105:281–8. 10.1159/000503583 31618740

[35] Zhang X Zhang P An L Sun N Peng L Tang W. Miltirone induces cell death in hepatocellular carcinoma cell through GSDME-dependent pyroptosis. Acta Pharm Sin B. (2020) 10:1397–413. 10.1016/j.apsb.2020.06.015 32963939 PMC7488361

[36] Song W Ma YY Miao S Yang RP Zhu Y Shu D. Ming, pharmacological actions of miltirone in the modulation of platelet function. Acta Pharmacol Sin. (2019) 40:199–207. 10.1038/s41401-018-0010-1 29795134 PMC6329759

[37] Wang H Gu J Hou X Chen J Yang N Liu Y. Anti-inflammatory effect of miltirone on inflammatory bowel disease via TLR4/NF-κB/IQGAP2 signaling pathway. Biomed Pharmacother. (2017) 85:531–40. 10.1016/j.biopha.2016.11.061 27903427

[38] Zhang L Zhang H Li X Jia B Yang Y Zhou P. Miltirone protects human EA.hy926 endothelial cells from oxidized low-density lipoprotein-derived oxidative stress via a heme oxygenase-1 and MAPK/Nrf2 dependent pathway. Phytomedicine. (2016) 23:1806–13. 10.1016/j.phymed.2016.11.003 27912883

[39] Yan W Guoping Y Chengxian G Qi P Ranran Z Lu H. Plasma double-peak phenomenon following oral administration. Chin J Clin Pharma Ther. (2014) 19:341–5.

