# Supplementary material for: Tentative exploration of pharmacodynamic substances: Pharmacological effects, chemical compositions, and multi-components pharmacokinetic characteristics of ESZWD in CHF-HKYd rats
Source: Front Cardiovasc Med. 2022 Sep 14;9:913661. doi: 10.3389/fcvm.2022.913661 (PMC9515952; doi:10.3389/fcvm.2022.913661)
Supplement: Supplementary file 1 [file Data_Sheet_1.docx]

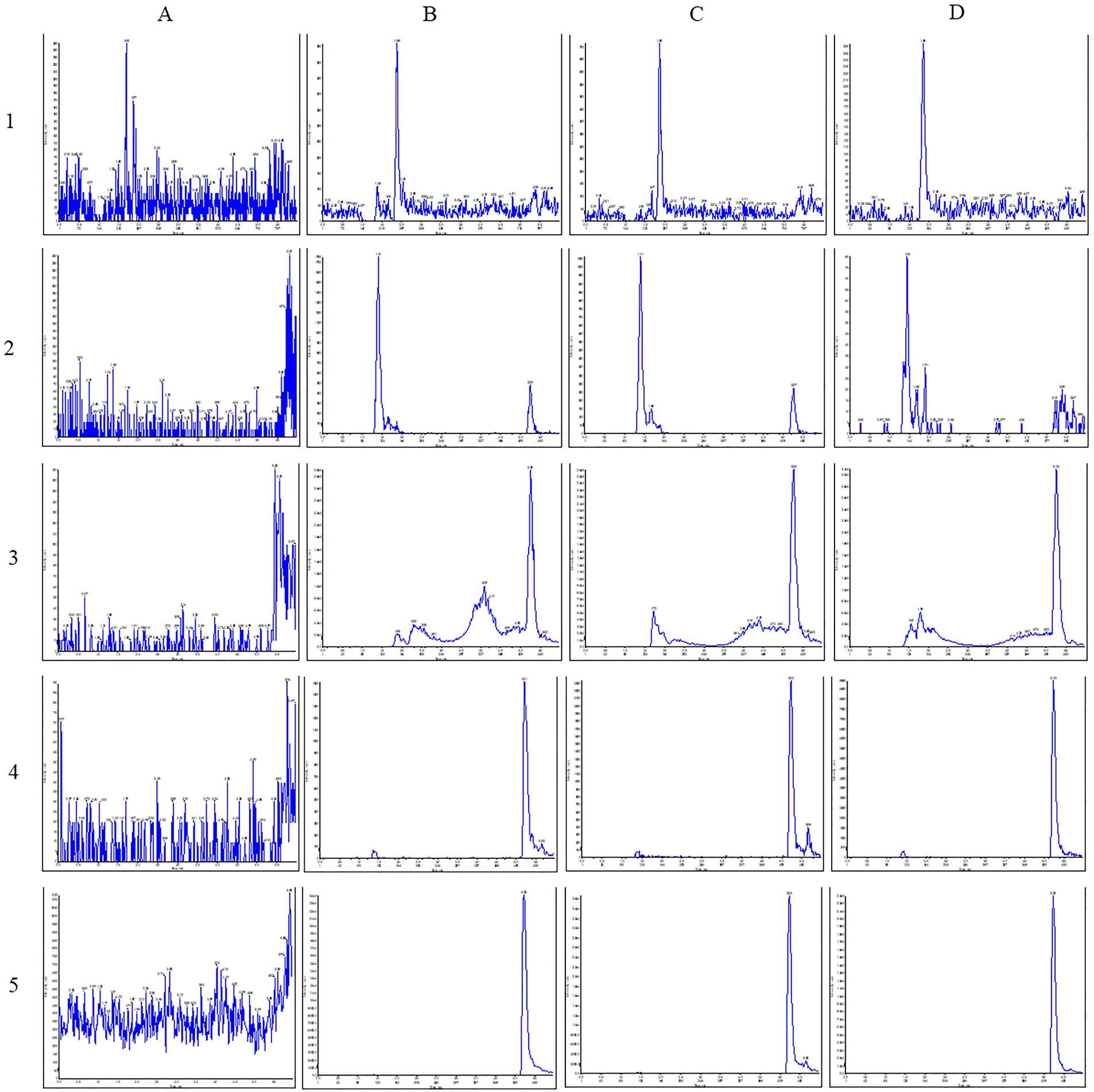


Figure 1 UPLC-MS/MS specific chromatograms of four compounds and Tanshinone IIA in rat serum

1. blank plasma; (B) four compounds and Tanshinone IIA (IS); (C) blank serum spiked with salsolinol, aconitine, paeoniflorin, miltrione and IS (1-8); (D) serum compounds and IS after oral administration of ESZWD.

Table 1 Precision of four compounds in rat serum (n = 5)

|  |  | Interday | | Interday | |
| --- | --- | --- | --- | --- | --- |
| Compounds | Added (ng/mL) | Mean ± SD (ng/mL) | RSD (%) | Mean ± SD (ng/mL) | RSD (%) |
| Salsolinol | 10 | 10.12±0.89 | 8.80 | 10.19±0.38 | 3.69 |
|  | 20 | 21.76±0.5 | 2.30 | 20.99±0.31 | 1.46 |
|  | 100 | 106.9±6.9 | 6.46 | 104.9±4.84 | 4.62 |
|  | 750 | 768.2±65.29 | 8.50 | 728.1±32.54 | 4.47 |
| Aconitine | 0.4 | 0.4305±0.01 | 2.02 | 0.4455±0.02 | 3.97 |
|  | 1 | 1.059±0.02 | 1.42 | 1.05±0.03 | 3.15 |
|  | 10 | 10.77±0.75 | 7.01 | 10.02±0.49 | 4.89 |
|  | 150 | 140.9±9.44 | 6.69 | 160.8±2.49 | 1.55 |
| Paeoniflorin | 0.5 | 0.4981±0.02 | 3.23 | 0.5177±0.04 | 8.05 |
|  | 1 | 1.056±0.05 | 5.10 | 1.017±0.08 | 7.43 |
|  | 10 | 10.70±0.51 | 4.74 | 10.04±0.82 | 8.22 |
|  | 150 | 155.9±6.82 | 4.38 | 151.8±11.99 | 7.89 |
| Miltrione | 0.4 | 0.3398±0.02 | 4.88 | 0.4145±0.03 | 6.74 |
|  | 1 | 1.068±0.04 | 3.37 | 1.024±0.07 | 6.68 |
|  | 10 | 10.55±0.75 | 7.15 | 9.911±0.15 | 1.49 |
|  | 150 | 131.3±4.2 | 3.20 | 137.6±3.82 | 2.77 |

Table 2 Accuracy of four compounds in rat serum (n = 5)

| Compounds | Added (ng/mL) | Mean ± SD (ng/mL) | RSD (%) |
| --- | --- | --- | --- |
| Salsolinol | 10 | 101.9±8.22 | 8.07 |
|  | 20 | 106.5±4.27 | 4.01 |
|  | 100 | 106.7±6.84 | 6.41 |
|  | 750 | 100.1±8.66 | 8.64 |
| Aconitine | 0.4 | 109.8±3.49 | 3.18 |
|  | 1 | 105.4±2.53 | 2.40 |
|  | 10 | 113.1±2.29 | 2.02 |
|  | 150 | 85.8±1.83 | 2.13 |
| Paeoniflorin | 0.5 | 98.27±2.71 | 2.76 |
|  | 1 | 95.02±5.14 | 5.41 |
|  | 10 | 92.37±1.82 | 1.97 |
|  | 150 | 92.60±5.21 | 5.62 |
| Miltrione | 0.4 | 89.96±7.13 | 7.92 |
|  | 1 | 104.0±8.9 | 8.56 |
|  | 10 | 103.7±8.97 | 8.65 |
|  | 150 | 87.31±2.83 | 3.25 |

Table 3 Matrix effect of four compounds in rat serum (n = 5)

| Compounds | Added (ng/mL) | Mean ± SD (ng/mL) | RSD (%) |
| --- | --- | --- | --- |
| Salsolinol | 20 | 99.26±2.53 | 2.55 |
|  | 100 | 90.91±7.81 | 8.59 |
|  | 750 | 99.08±9.09 | 9.17 |
| Aconitine | 1 | 94.3±4.16 | 4.41 |
|  | 10 | 114.9±8.45 | 7.35 |
|  | 150 | 87.2±2.02 | 2.31 |
| Paeoniflorin | 1 | 86.01±8.20 | 9.53 |
|  | 10 | 99.56±10.79 | 10.83 |
|  | 150 | 99.58±9.76 | 9.80 |
| Miltrione | 1 | 93.85±13.85 | 14.76 |
|  | 10 | 112.3±6.07 | 5.40 |
|  | 150 | 114.3 ± 14.71 | 12.86 |

Table 3 Stability of four compounds in rat serum (n = 5)

|  |  | Room temperature | | Automatic sampler | | Multigelation | | Long-term freeze | |
| --- | --- | --- | --- | --- | --- | --- | --- | --- | --- |
| Compounds | Added (ng/mL) | Mean ± SD (ng/mL) | RSD (%) | Mean ± SD (ng/mL) | RSD (%) | Mean ± SD (ng/mL) | RSD (%) | Mean ± SD (ng/mL) | RSD (%) |
| Salsolinol | 20 | 21.07±0.74 | 3.50 | 20.68±0.61 | 2.95 | 21.15±0.54 | 2.56 | 20.9±1.2 | 5.73 |
|  | 100 | 112.2±4.13 | 3.68 | 111.3±2.63 | 2.36 | 110.7±1.34 | 1.21 | 103.9±6.16 | 5.93 |
|  | 750 | 700.5±49.6 | 7.08 | 716.3±22.1 | 3.09 | 727.6±68.05 | 9.35 | 685.1±43.66 | 6.37 |
| Aconitine | 1 | 1.049±0.05 | 4.31 | 1.087±0.12 | 10.99 | 1.125±0.11 | 9.80 | 1.082±0.05 | 4.94 |
|  | 10 | 9.558±0.29 | 3.06 | 9.053±0.24 | 2.66 | 9.042±0.48 | 5.32 | 10.05±0.97 | 9.61 |
|  | 150 | 163.7±5.25 | 3.21 | 162.6±9.72 | 5.98 | 156.9±8.39 | 5.35 | 157.4±3.86 | 2.45 |
| Paeoniflorin | 1 | 1.046±0.06 | 5.78 | 1.053±0.02 | 2.13 | 1.012±0.07 | 7.34 | 1.010±0.08 | 7.59 |
|  | 10 | 10.18±0.72 | 7.06 | 10.51±0.98 | 9.33 | 10.41±0.73 | 7.04 | 10.19±0.97 | 9.50 |
|  | 150 | 160.7±5.68 | 3.53 | 160.2±6.15 | 3.84 | 162.2±9.25 | 5.70 | 149.6±11.70 | 7.82 |
| Miltrione | 1 | 1.024±0.15 | 14.50 | 1.109±0.15 | 13.60 | 1.065±0.06 | 5.46 | 1.012±0.09 | 9.23 |
|  | 10 | 10.03±0.92 | 9.20 | 10.22±0.73 | 7.16 | 9.899±0.47 | 4.73 | 9.999±0.15 | 1.46 |
|  | 150 | 133.1±5.00 | 3.76 | 134.1±4.56 | 3.41 | 136.5±5.3 | 3.88 | 132.8±5.8 | 4.37 |

Table 4 Serum NT-proBNP level of different time in CHF-HKYd rats

| Time (h) | NT-proBNP (pg/mL) |
| --- | --- |
| 0.083 | 133.9814 |
| 0.17 | 118.5356 |
| 0.33 | 95.89687 |
| 0.5 | 89.19747 |
| 0.75 | 74.59917 |
| 1 | 112.9669 |
| 2 | 119.0605 |
| 4 | 125.9014 |
| 6 | 124.4105 |
| 8 | 109.8226 |
| 12 | 148.0687 |
| 24 | 137.4947 |
